# The Australian millipede *Dicranogonus pix* Jeekel, 1982 (Diplopoda, Polydesmida, Paradoxosomatidae): a species with and without paranota

**DOI:** 10.3897/zookeys.454.8625

**Published:** 2014-11-12

**Authors:** Robert Mesibov

**Affiliations:** 1 Queen Victoria Museum and Art Gallery, 2 Invermay Road, Launceston, Tasmania, Australia 7248

**Keywords:** Millipede, Diplopoda, Polydesmida, Paradoxosomatidae, Tasmania, Victoria, Bass Strait, Australia, biogeography

## Abstract

*Dicranogonus pix* Jeekel, 1982 occurs in Victoria and Tasmania, Australia, including the islands in eastern Bass Strait between the two States. There is only slight gonopod variation across this range, but *Dicranogonus pix* populations with and without paranota are separated in Bass Strait by the ca 50 km-wide gap between the Kent and Furneaux Groups of islands.

## Introduction

*Dicranogonus* was erected by Jeekel (1982) for *Dicranogonus pix* Jeekel, 1982, a small, dark paradoxosomatid with a simply forked gonopod. The new species had been collected two years earlier at three localities in eastern Victoria by the visiting Dutch specialist Dr C.A.W. Jeekel and his wife, A.M. Jeekel-Rijvers (Jeekel 1981).

Somewhat cryptically, Jeekel (1982: 209) wrote “The genus *Dicranogonus* has a second, as yet undescribed, species on the islands of the Furneaux group between Victoria and northeastern Tasmania”. Jeekel did not travel to the Furneaux Group during his time in Australia, and prior to his death in 2010, Jeekel did not publish any hints regarding where he had seen specimens of a second *Dicranogonus* species, or how it differed from *Dicranogonus pix*.

In 1984, Jeekel proposed that Victoria had been a centre of dispersal for *Dicranogonus*, *Pogonosternum* Jeekel, 1965 and *Somethus* Chamberlin, 1920 (Jeekel 1984: 44). At the time, Jeekel was evidently unaware of the occurrence of *Dicranogonus* on the Tasmanian mainland, writing “The distribution of *Dicranogonus* seems to indicate that migration from Victoria southward towards Tasmania along a north-eastern route was blocked south of the Furneaux Group” (Jeekel 1984: 44).

In a later publication, however, Jeekel mentioned that *Dicranogonus* also occurs in Tasmania (Jeekel 2006: 82). I am not certain whether he was referring to the Furneaux Group, which is politically part of Tasmania, or to the northeast Tasmanian mainland, from which I had earlier reported the presence of *Dicranogonus* (Mesibov and Churchill 2003).

As shown below, *Dicranogonus* occurs in two strikingly different forms: one in Victoria and northeast Bass Strait with obvious paranota, and one without paranota in southeast Bass Strait (in the Furneaux Group) and on the northeast Tasmanian mainland. In this paper I treat both forms as *Dicranogonus pix*, and in the Discussion section I explain the reasons for this taxonomic decision.

## Methods

All specimens I examined are in registered specimen lots in Australian repositories and are listed in the accompanying data table. Latitude/longitude figures in the table are given with the WGS84 datum together with my estimate of the spatial uncertainty (Darwin Core CoordinateUncertaintyInMeters).

Colour photomicrographs of specimens in 80% ethanol were taken with a Canon EOS 1000D digital SLR camera mounted on a Nikon SMZ800 binocular dissecting microscope equipped with a beam splitter. Colour images used in the figures are focus-stacked composites prepared with Zerene Stacker 1.04 software. Grayscale images of gonopod telopodites temporarily mounted in 1:1 glycerol:water were captured as screenshots from the output of a 1.3 megapixel digital video eyepiece camera mounted in one ocular tube of a Tasco LMSMB binocular microscope. The screenshots were edited with GIMP 2.8 software to remove background highlights and artefacts. Measurements were made in all cases to the nearest 0.1 mm with eyepiece grids and reference scales. The SEM images in Fig. 5 are of an isolated body ring which was air-dried and sputter-coated with gold before examination and image capture with an FEI Quanta 600 ESEM operated in high vacuum mode. (Another version of Fig. 5B appeared as Fig. 2B in Mesibov (2009), where the specimen was identified as “*Dicranogonus* sp.”.) Base maps were generated with ArcView 3.2 GIS software.

Abbreviations below and in the accompanying data table: AM = Australian Museum, Sydney, New South Wales, Australia; DPIPWE = New Town Laboratories, Department of Primary Industries, Parks, Water and Environment, New Town, Tasmania, Australia; NBC = Naturalis Biodiversity Center, Leiden, Netherlands; NMV = Museum Victoria, Melbourne, Victoria, Australia; NSW = New South Wales, Australia; QVM = Queen Victoria Museum and Art Gallery, Launceston, Tasmania, Australia; Tas = Tasmania, Australia; TMAG = Tasmanian Museum and Art Gallery, Hobart, Tas; Vic = Victoria, Australia.

## Results

### Order Polydesmida Pocock, 1887 Suborder Strongylosomatidea Brölemann, 1916 Family Paradoxosomatidae Daday, 1889 Subfamily Australiosomatinae Brölemann, 1916 Tribe Antichiropodini Brölemann, 1916

#### Dicranogonus

Taxon classificationAnimaliaPolydesmidaParadoxosomatidae

Jeekel, 1982

Dicranogonus : Jeekel 1982: 208; 2006: 82. Shelley et al. 2000: 97. Nguyen and Sierwald 2013: 1155.

##### Type species.

*Dicranogonus pix* Jeekel, 1982, by original designation.

#### Dicranogonus pix

Taxon classificationAnimaliaPolydesmidaParadoxosomatidae

Jeekel, 1982

**Figure 1.**
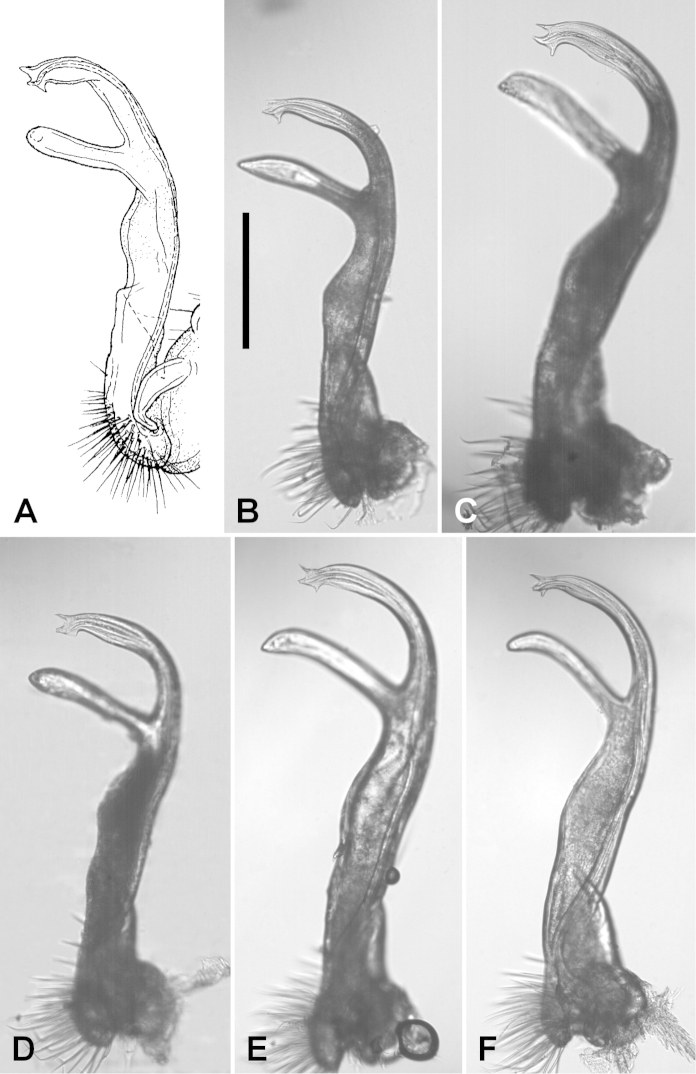
*Dicranogonus pix* Jeekel, 1982, right gonopod telopodite, anterior views. **A** Holotype, from Fig. 4 in Jeekel (1982), used with permission **B** NMV K-10010 **C** AM KS.105124 **D** AM KS.94201 **E** QVM 23:46456 **F** QVM 23:21876. Scale bar for **B–F** = 0.25 mm, with focus on solenomere tip. See Fig. 3A for mapped localities.

**Figure 2.**
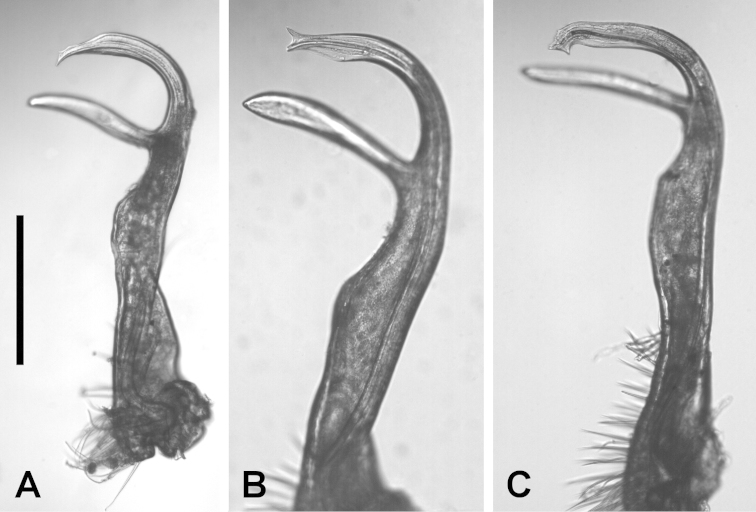
*Dicranogonus pix* Jeekel, 1982, right gonopod telopodite, anterior views. **A** NMV K-10011 **B** TMAG J3286 **C** QVM 23:40085. Scale bar for **A–C** = 0.25 mm, with focus on solenomere tip. See Fig. 3A for mapped localities.

**Figure 3.**
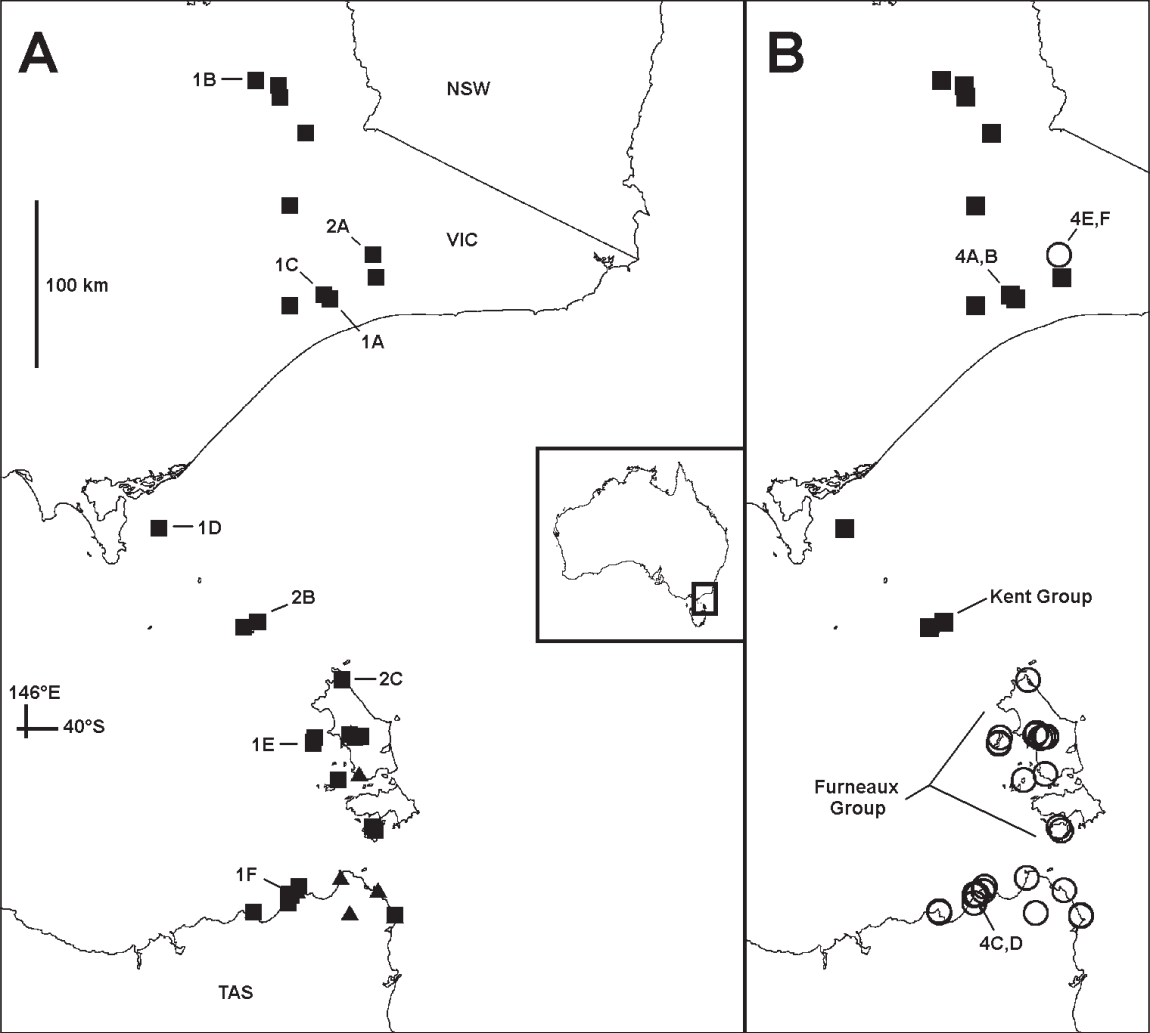
*Dicranogonus pix* Jeekel, 1982, known localities as of 17 September 2014. **A** Localities with males (squares) and with females only (triangles); labels indicate localities of males with gonopods imaged in Figs 1 and 2; **B** Specimens with paranota (filled squares) and without paranota (unfilled circles); labels indicate localities of specimens with midbody rings imaged in Fig. 4. Mercator projection. Inset shows Australia with map area of **A** (rectangle).

**Figure 4.**
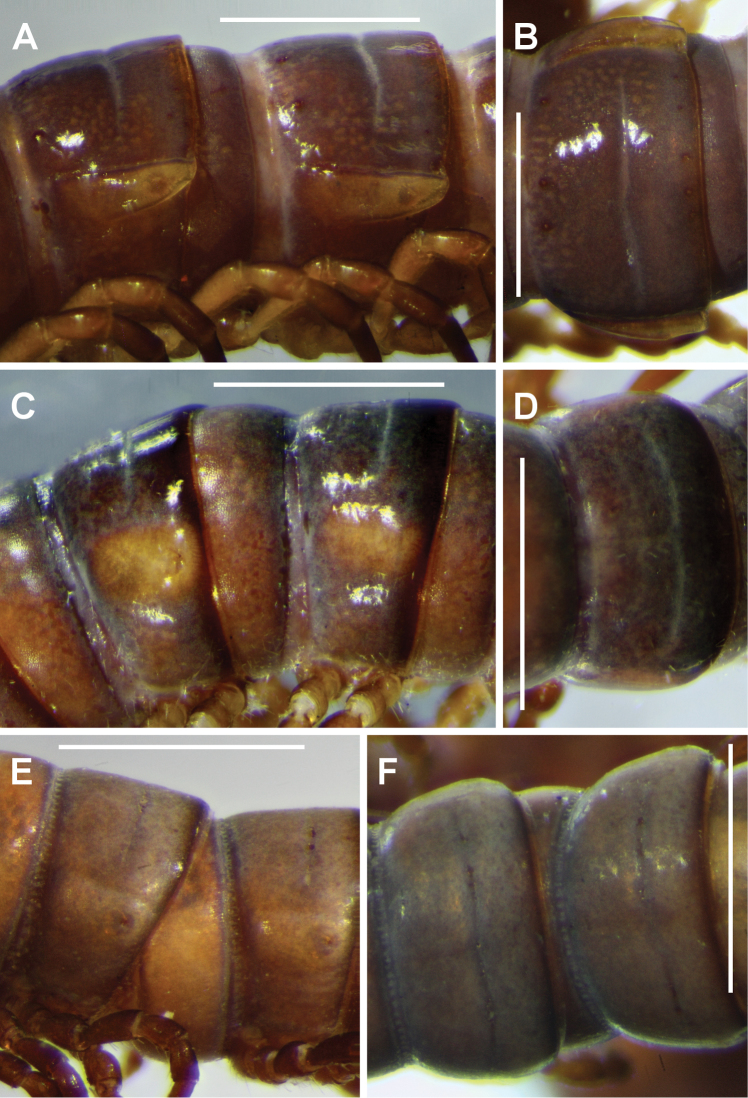
*Dicranogonus pix* Jeekel, 1982, males; **A**, **C**, **E** left lateral views of midbody rings **B**, **D**, **F** dorsal views of midbody rings. **A**, **B** AM KS.105124 **C**, **D** QVM 23:21875 **E**, **F** NMV K-10011. Scale bars = 1.0 mm. See Fig. 3B for mapped localities.

**Figure 5.**
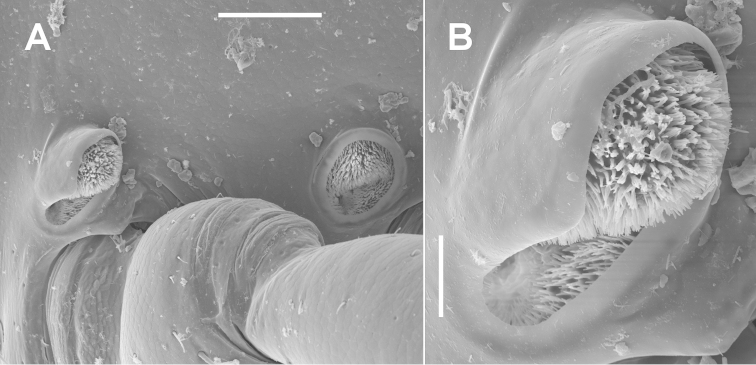
*Dicranogonus pix* Jeekel, 1982, male ex QVM 23:15170. **A** Left lateral view of midbody spiracles **B** Anterior spiracle. Scale bars: **A** = 0.1 mm, **B** = 0.025 mm.

Figs 1 , 2 , 3 , 4 , 5

Dicranogonus pix Jeekel 1982: 209; Fig. 4 (p. 206). Shelley et al. 2000: 97. Mesibov 2004: 42; 2009: Fig. 2B (p. 534). Nguyen and Sierwald 2013: 1155.

##### Morphology.

*Gonopods.* The gonopod telopodite varies only slightly in details over the *Dicranogonus pix* range (Figs 1–3). There are two small tabs (Jeekel: “lappets”) on either side of the solenomere tip in the holotype, and one or both tabs (more often the basally directed one) are reduced or missing in some populations (Fig. 2). The most divergent male examined is from the northeast corner of Flinders Island in the Furneaux Group (Fig. 2C); the tip of the solenomere in this specimen is abruptly curved basally and the subapical process of the telopodite (Jeekel: “tibiotarsus”) is thinner and closer to the solenomere than in most males.

*Paranota.* In agreement with the original description of *Dicranogonus pix*, the diplosegments of a nearly topotypical male have obvious paranota (Figs 3, 4A, 4B). Similarly well-defined paranota are present on almost all *Dicranogonus* specimens from eastern Victoria and small islands in the northeast portion of Bass Strait. In contrast, all specimens from islands in the southeast portion of Bass Strait (i.e., the Furneaux Group) and the Tasmanian mainland lack paranota (Figs 3, 4C, 4D), although the paranotal area on diplosegments is usually marked by lighter colour, and on some rings there is a very slight lateral bulge at the level of the ozopore.

The only exceptions to this simple geographical pattern in the material examined are three males and two females lacking paranota from the Buchan district in eastern Victoria, collected in 1907 (Figs 3, 4E, 4F).

*Other characters.* I add here only a few minor details to the very clearly written, 1600-word description by Jeekel (1982) of the typical *Dicranogonus pix*. Spiracles on diplosegments located just above and anterior to leg bases (Fig. 5A); anterior spiracle (Fig. 5B) ovoid with long axis nearly vertical, anterodorsal portion of rim extended as thin cowl and directed slightly posteriorly; posterior spiracle nearly round, rim slightly raised and rounded; anterior and posterior spiracular filters composed of numerous thin, forked tabs with blunt tips (Fig. 5B), the dorsal half of the filter produced in the posterior spiracle and emergent in the anterior spiracle. Paranota on diplosegments well-defined to ring 16, then progressively diminishing to a very slight lateral bulge on ring 19. Spinnerets in square array.

##### Biogeography.

*Dicranogonus pix* and *Notodesmus scotius* Chamberlin, 1920 are the only Polydesmida so far known to occur naturally on both sides of Bass Strait (see *Notodesmus scotius* distribution records and KML file at http://www.polydesmida.info/millipedesofaustralia/localities.html). The *Notodesmus scotius* material I have examined is uniform throughout the species’ range in Tasmania, Victoria and southeast New South Wales, and I have not detected any morphological discontinuity in *Notodesmus scotius* in Bass Strait.

For other poorly vagile animals with trans-Bass Strait distributions, I have not yet found any documentation of discontinuities congruent with the paranota/no-paranota divide in *Dicranogonus pix* between the Kent and Furneaux Groups. A possible evolutionary parallel is in the rhaphidophorid cricket genus *Cavernotettix* Richards, 1966. *Cavernotettix flindersensis* (Chopard, 1944) is known only from the Furneaux Group, and *Cavernotettix craggiensis* Richards, 1974 is known only from Craggy Island (ca 40 ha), located between the Kent and Furneaux Groups. (*Cavernotettix* records from the online *Atlas of Living Australia*, http://www.ala.org.au, accessed 17 September 2014.)

Within Victoria the known distribution of *Dicranogonus pix* is a zone ca 150 km long and up to 60 km wide, running north from East Gippsland over the Great Dividing Range, from near sea level to ca 600 m. On the Tasmanian mainland all but one of the locality records are less than ca 2 km from the sea, the exception being a 1964 collection from Gladstone, a small town. “Gladstone”, however, may only represent the nearest named place to a coastal collecting site on Ringarooma Bay, ca 15 km distant. I have collected *Notodesmus scotius*, but not yet *Dicranogonus pix*, in the dry eucalypt forests beginning ca 10 km inland from *Dicranogonus pix* localities along the northeast Tasmanian coast.

**Ecology.** Jeekel (1982: 212) wrote of *Dicranogonus pix* at the holotype and paratype localities in Victoria: “This elegant little creature was locally quite common, occurring numerously in the upper litter layer of the dry Eucalyptus forests, and, judging from the number of specimens seen, mass appearances may occasionally happen”. Adults have so far been collected in every month of the year except May, and in August 1998 I found a mixed *Notodesmus scotius*–*Dicranogonus pix* ‘mating swarm’ during the day in coastal heathland near Blackmans Lagoon in northeast Tasmania.

Surprisingly, *Dicranogonus pix* was missing from pitfall samples collected in coastal heathland within the *Dicranogonus pix* range in northeast Tasmania in 1986-88. The sampling was carried out by T.B. Churchill, who trapped paradoxosomatids (as by-catch) in three 9 × 9 m pitfall arrays (nine evenly spaced traps per array) located at each of four 90 × 90 m sampling sites, with the traps emptied once a month for 14 months (Mesibov and Churchill 2003). The traps yielded 9754 specimens of *Notodesmus scotius* and 116 specimens of an undescribed *Pogonosternum* species.

##### Type specimens.

Jeekel (1982: 209) lists the following type specimens for *Dicranogonus pix*, all collected on 14 November 1980 by Dr Jeekel and A.M. Jeekel-Rijvers:

Holotype male: “Sta. 86. 4 km ESE Bruthen... Eucalyptus forest, State forest, under logs” [My location estimate for the type locality near Bruthen, Vic is 37°43'18"S 147°52'24"E ±1 km, probably along the Bruthen-Buchan Road.]

Paratypes: 3 males, 6 females, details as for holotype; 38 males, 29 females, “Sta. 85. 13 km SE Buchan... Eucalyptus forest, State forest, under logs” [37°36'S 148°11'E ±2 km, probably along a forest road]; 46 males, 73 females, “Sta. 87. Mt Taylor, 11 km NNW Bairnsdale...fragment of Eucalyptus forest, along roadside between grassland, under logs and litter” [37°45'28"S 147°35'55"E ±1 km, possibly along Bullumwaal Road].

*Dicranogonus* samples from the three localities listed above have recently been located in the Naturalis Biodiversity Center (K. van Dorp, in litt., 17 September 2014), following a long period during which their location was uncertain. The three samples, which I have not examined, presumably contain the holotype and the published paratypes.

## Discussion

The presence or absence of well-defined paranota on diplosegments is usually a genus-level character in Polydesmida. It is remarkable that both character states are found, with no obvious intermediates, in Australian paradoxosomatid specimens with no consistent, diagnosable differences in the gonopod telopodite between the forms with and without paranota (Fig. 1). The genus *Dicranogonus* (as diagnosed on gonopod form) thus offers an extreme example of the ‘diphasic evolution’ posited by Hoffman (1981) for Polydesmida. He observed that in many lineages, gonopods vary greatly with only minor accompanying variation in body form, while in a small minority of lineages the opposite is true.

Consistent, diagnosable gonopod variations have long been the basis of species-level taxonomy in Polydesmida. Without such variations, species delimitation on purely morphological evidence is hard to justify, especially if the taxonomist has only a limited number of specimens from an incomplete sampling of the distribution of the genus. In the case of *Dicranogonus*, however, there is abundant material from localities across the genus range. The geographical pattern for presence and absence of paranota (Fig. 3) is almost perfectly allopatric. It is tempting to delimit *Dicranogonus* species, as Jeekel may have done informally (see Introduction), on paranotal presence/absence and on geography.

However, the five specimens from Buchan (NMV K-10011; Figs 2A, 4E, 4F), in the heart of the eastern Victorian distribution of *Dicranogonus*, also lack paranota. They were collected in 1907 by the naturalist J.A. Leach (http://en.wikipedia.org/wiki/John_Albert_Leach), at the time the district inspector of schools for East Gippsland, Victoria, and I regard it as unlikely that the sample was mislabelled or that the specimens represent descendants of introduced *Dicranogonus* from Tasmania or the Furneaux Group. A more plausible interpretation is that loss of paranota has occurred at least twice in the *Dicranogonus* lineage. Loss occurred in an ancestral population which had reached the Furneaux Group or mainland Tasmania, and also in the ancestor of the paranota-less Buchan specimens.

The timing of these losses might be estimated from future genetic work on *Dicranogonus*. For current taxonomic purposes, I am satisfied that all material I examined can be assigned to *Dicranogonus pix*, which is readily diagnosed on gonopod form and body size and colour.

## Supplementary Material

XML Treatment for Dicranogonus

XML Treatment for Dicranogonus pix

## Supplementary materials

Supplementary material 1 Data table Data type: CSV file. Explanation note: Known specimen lots of *Dicranogonus pix* Jeekel, 1982 as of 17 September 2014. This dataset is made available under the Open Database License (http://opendatacommons.org/licenses/odbl/1.0/). The Open Database License (ODbL) is a license agreement intended to allow users to freely share, modify, and use this Dataset while maintaining this same freedom for others, provided that the original source and author(s) are credited.Robert Mesibov
